# Censored Data Analysis Reveals Effects of Age and Hepatitis C Infection on C-Reactive Protein Levels in Healthy Adult Chimpanzees (*Pan troglodytes*)

**DOI:** 10.1155/2013/709740

**Published:** 2013-02-27

**Authors:** John J. Ely, Tony Zavaskis, M. Lon Lammey

**Affiliations:** Alamogordo Primate Facility, Building 1303, P.O. Box 956, Holloman AFB, NM 88330-0956, USA

## Abstract

C-reactive protein, a conserved acute-phase protein synthesized in the liver and involved in inflammation, infection, and tissue damage, is an informative biomarker for human cardiovascular disease. Out of 258 captive adult common chimpanzees (*Pan troglodytes*) assayed for CRP, 27.9% of the data were below the quantitation limit. Data were analyzed by the Kaplan-Meier method and results compared to other methods for handling censored data (including deletion, replacement, and imputation). Kaplan-Meier results demonstrated a modest age effect and a strong effect of HCV infection in reducing CRP but did not allow inference of reference intervals. Results of other methods varied considerably. Substitution schemes differed widely in statistical significance, with estimated group means biased by the size of the substitution constant, while inference of unbiased reference intervals was impossible. Single imputation gave reasonable statistical inferences but unreliable reference intervals. Multiple imputation gave reliable results, for both statistical inference and reference intervals, and was comparable to the Kaplan-Meier standard. Other methods should be avoided. CRP did not predict cardiovascular disease, but CRP levels were reduced by 50% in animals with hepatitis C infection and showed inverse relationships with 2 liver function enzymes. Results suggested that hsCRP can be an informative biomarker of chronic hepatic dysfunction.

## 1. Introduction

C-reactive protein (CRP), a phylogenetically highly conserved protein, has become an important biomarker of acute inflammation and tissue damage in humans [1–3]. CRP is an important biomarker for many aspects of health and disease, including cardiovascular disease, type 2 diabetes, and chronic renal disease and is a predictor of all-cause mortality [2, 4–7]. CRP is synthesized by hepatocytes when induced by cytokines including IL-6 [1, 4]. Circulating plasma levels can rise during the acute-phase response to inflammation, infection, or trauma by 10,000-fold and decrease just as rapidly [1, 2, 8]. CRP has many biological functions related to the recognition and clearance of foreign pathogens and damaged host cells, binding chromatin and small nuclear ribonucleoproteins, which suggested a role in clearance of debris due to apoptosis and necrosis [2, 4, 8]. CRP stimulates the classical complement pathway [1, 2, 4]. Its activation by the same Fc receptors used by IgG, and its earlier response to infection, suggested a role in inducing an adaptive immune response [4]. 

CRP is also involved in the development of atherosclerotic lesions and plaque disruption [8, 9]. Epidemiological evidence for the role of inflammation in the etiology of coronary heart disease, myocardial infarction, and peripheral vascular disease has indicated a role for CRP as a biomarker for cardiovascular disease [8, 10–12]. CRP was a better overall predictor of cardiovascular events than LDL cholesterol, conferred additional prognostic information to Framingham risk scores, and reduce all-cause mortality among otherwise asymptomatic humans [10, 13–15]. In a 16-year long prospective study, high levels of hsCRP (>3 mg/L) were associated with a 2-fold increased risk of all-cause mortality relative to low hsCRP levels (<1 mg/L) in healthy humans [7]. Meta-analyses have consistently demonstrated a strong association (OR ≥ 2.0) between elevated CRP levels and major coronary events, [16, 17] although there remains some debate over the most informative cut-off values. A 7-year followup study found that very high levels of hsCRP (>10 mg/L) were more strongly associated with risk of clinical cardiovascular disease and with all-cause mortality, compared to merely high (3–10 mg/L) levels [5, 11, 18]. 

Cardiovascular disease (CVD) is the primary cause of morbidity and mortality in captive chimpanzees [19–25]. This suggested the potential utility of CRP as a biomarker of CVD in aging chimpanzees, not unlike other biomarker studies [19, 20]. Similarly, hepatitis C has been associated with reduced CRP levels in humans [26–28]. Historical use of chimpanzees in studies of viral hepatitis [29] suggested a role for hsCRP as a biomarker of hepatic damage. There was one earlier study of CRP in chimpanzees [30], but results were limited due to small sample size (*N* = 37) and wide age range (<1 yr to 44 yr old) with subadults not distinguished from adults (>10 yr old); sex differences were not evaluated, and associations with CVD or hepatitis infection were not investigated [30]. Finally, out-dated “normal ranges” were uncritically defined, rather than using reliable methods from human laboratory medicine [20, 31]. Therefore, it was of interest to evaluate CRP as a potential biomarker for CVD and hepatic dysfunction and to define reference intervals in a large captive population of chimpanzees. 

## 2. Methods

### 2.1. Colony

At the time of this cross-sectional study, the Alamogordo Primate Facility (APF) housed 258 adult research-reserve chimpanzees (*Pan troglodytes*) primarily descended from the West African *P.t. verus* subspecies [32]. The APF animal program and facilities were fully accredited by AAALAC, with animals maintained in same-sex group housing, to comply with the NIH breeding moratorium [33]. The study was fully approved by the ACUC and conducted in accordance with the *Guide for the Care and Use of Laboratory Animals* [34]. Animals were maintained in socially compatible groups in indoor dens (180 ft^2^, 9.5 ft high) with radiant heated floors and air conditioning, 24 hr access to outdoor dens (242 ft^2^), and weekly access to outdoor play yards (802 ft^2^). Diet consisted of commercial primate chow (Purina Lab Diet Monkey Diet Jumbo 5LR2) plus daily fresh fruits and vegetables delivered to simulate naturalistic foraging opportunities. All animals received a complete physical examination and health assessment annually [20]. Prior (<2001) experimental exposures resulted in 61 animals with HCV infection (HCV antibody and PCR positive, >103 HCV genome equivalents/mL), and the remainder (196) not infected (Table 1). Seven subadults (<10 years old) on-site were excluded from analyses [19, 20, 35]. 

### 2.2. hsCRP Assay

All 258 adults (116 female, 142 male) were assayed for hsCRP. Ten mL whole blood samples were collected in serum separator tubes, separated by low-speed centrifugation, transferred into sterile vials, and shipped overnight on ice to a clinical reference laboratory (Tricore Industries, Albuquerque, NM). Samples were tested for hsCRP with a rate turbidimetric immunoassay using polyclonal goat and mouse anti-CRP antibody bound to latex particles [36, 37]. The detection limit (DL, sometimes mistakenly called analytical sensitivity) was 0.06 mg/L, below the level recommended to predict cardiovascular events [11]. The reporting or quantitation limit (QL), below which non-linearity and high error relative to signal render single reportable numbers unreliable, was 0.4 mg/L, well within reported limits (mean QL = 8.6∗DL) for other hsCRP assays [38–40]. Unreliable estimates below the QL threshold are termed left censored and require use of statistical methods designed for analysis of left-censored data [39]. 

To validate the use of the human CRPH assay, chimpanzee CRP DNA sequence (NCBI entry XM_001170732) and its deduced amino acid sequence were aligned with the human homologues (NCBI NM_000567) [28] and species-specific differences identified with ClustalX2 [41, 42]. The effects of species-specific amino acid substitutions on the structure and function of chimpanzee CRP, relative to the human protein, were inferred using Polyphen-2, an on-line protein structure/function server, based on biochemical and comparative principles [43, 44]. Chimpanzee CRP mRNA showed 9 nucleotide (nt) substitutions and 2 indels relative to the human sequence. Of these, 3 substitutions (bases 367, 374 and 601) occurred in the actual protein coding region, 2 of which resulted in non-synonymous substitutions. The first (nt position 367) involved an A/G transition at the second codon position and resulted in a non-synonymous amino acid change, from aspartic acid in humans to glycine in chimpanzees (D88G). The second (nt 374) involved an A/G transition at the third wobble codon position and was silent. The third (nt 601) involved an A/G transition at the second codon position and resulted in a non-synonymous amino acid change, from glycine in humans to glutamic acid in chimpanzees (G166E). Both coding mutations occurred in the pentaxin domain, D88G in a beta-pleated sheet, and G166E in an alpha helix [45]. Neither occurred at a critical site in the mature protein, such as calcium binding sites or disulfide bonds [3, 45]. Structural predictions using PolyPhen [43, 44] indicated that both mutations were benign (D88G, Polyphen score = 0.000; G166E, Polyphen score = 0.001). The mouse CRP protein sequence (Uniprot P14847) exhibited 70 amino acid differences (29.4%) from the human protein sequence, including alanine at mutated position 166, compared to only 2 differences (0.9%) between humans and chimpanzees. In short, there was no evidence that either of the 2 non-synonymous mutations would alter the binding efficiency or reduce the reliability of the CRPH assay in chimpanzees. 

### 2.3. Statistical Methods

**Figure 1:**
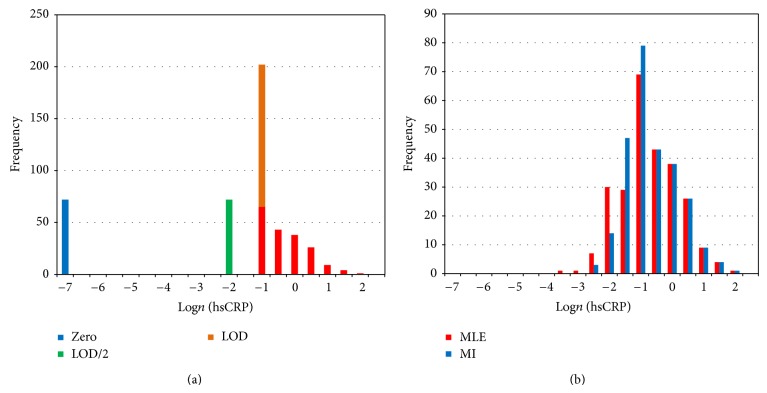
(a) Frequency histogram of hsCRP levels from the case-wise deletion and 4 substitution (QL, QL/2, zero) datasets. (b) Frequency histograms of hsCRP from the MLE and MI datasets.

All statistical analyses were performed on SYSTAT Version 11.0 (SYSTAT Software, Inc., Richmond, CA). We hypothesized that hsCRP levels would be elevated in unhealthy versus healthy animals, and that HCV-infected animals would have reduced CRP levels, compared to uninfected animals [20, 28, 31]. The Kaplan-Meier non-parametric product-limit method (KM) was used to analyze left-censored data, after “flipping” the data by subtraction from a large constant, resulting in a right censored dataset [39, 46, 47]. Choice of constant is arbitrary but does not affect results [39]. Statistical significance was assessed by Tarone-Ware *X* ^2^ statistics, which are intermediate in value between log-rank and Wilcoxon statistics [48]. KM was considered the inferential standard of reference for the other statistical methods described below. KM does have some limitations. Estimated means are unreliable due to extensive skew of non-normally distributed survival data [39]. The median is more robust to skew and outliers [49]. But depending on the pattern and extent of censoring, the exact median cannot always be estimated by KM. Therefore, KM does not necessarily allow reliable estimation of effect sizes. Finally, unless censored data are replaced by probable values, the bottom end of the distribution remains missing, and inference of reliable reference intervals is not possible. Therefore, other statistical methods for analyzing censored data (substitution, single MLE imputation, and multiple imputation) were used to replace censored observations with probable values. Those results were compared to the KM standard and also used to estimate reference intervals. ANOVA was used to analyze continuous data in the substitution and imputation datasets, with statistical significance determined by omnibus F statistics and single degree-of-freedom focused comparisons [50, 51]. Age and sex were used as covariates because they influence the distribution of health, disease, and CRP levels in populations [3, 19–21, 52]. The Shapiro-Wilks goodness-of-fit test rejected the assumption of a Normal (Gaussian) distribution (*W* = 0.677, *P* < 0.000). Substitution datasets could not be normalized, due to clustering of identical substituted values at the bottom of the distribution (Figure 1(a)). The case-deletion, MLE, and MI datasets were normalized with a log_*n*_ transformation [11, 18, 53–55]. Transformation efficacy was confirmed visually (Figure 1(b)) and with coefficient-based tests (G^1^/skew and G^2^/kurtosis) [31, 56]. Outliers were detected and eliminated using the robust interquartile method [49]. Categorical data were analyzed with contingency table methods, with statistical significance determined by likelihood ratio *G* ^2^ statistics [57, 58]. 

### 2.4. Data Imputation

hsCRP levels were assayed for all 258 adult chimpanzees (≥10 yr old). Of these, 185 adults (88 females, 98 males) had reliably quantified hsCRP levels, while 72 animals (28 females, 44 males) had unreliable (sub-threshold) levels (Table 1). This degree of censoring is considered moderate [59]. These data involved type I censoring, characterized by a fixed cut-off value at the quantitation limit (QL) and a variable number of censored observations [59]. (Type II censoring involves a variable cut-off value but a fixed number of censored observations [59]. For example, an LD_50_ study would terminate after observing 50% mortality, regardless of the survival times of the remaining study subjects [60]. Type II censoring will not be further discussed.) We used Little's [61] missing completely at random (MCAR) test statistic and contingency table methods [57, 58] to test for nonrandom patterns of hsCRP data missingness (nondetected or observed) relative to other factors (age, sex, hepatitis C infection, cardiovascular disease, and health status). 

Many scientific fields, from medicine to zoology, encounter an otherwise peculiar feature of data, wherein some observations fail to exceed a lower (or exceed an upper) threshold of detection [20, 39, 46, 47]. Such unobserved data are called censored, and the observations are called nondetects. Censoring that occurs relative to a lower limit is called left censored. In environmental epidemiology, many environmental pollutants occur at such minute concentrations that they cannot be reliably measured [62]. Left censoring raises the question whether nondetects represented true zero concentrations, or whether they were very small, but nonzero quantities only reported as zero, due to technical constraints [39]. Censoring that occurs relative to an upper limit is called right censored. Data on patients in prospective epidemiological studies who survive to the last date of followup are right censored, because the event of interest (such as mortality or cancer remission) was not observed during the study's timeframe [63]. Patients alive at last observation will have a minimum survival duration. Similarly, very high biomarker concentrations will exceed the limit of linearity (LOL) of the concentration/signal response, resulting in a minimum concentration [38, 39]. The presence of lower or upper limits result in informative or non-ignorable censoring [64]. Although exact values remain unknown, censored data are informative because they are known to be less than (or greater than) some lower (or upper) threshold [19, 39]. The class of statistical methods developed to handle this troublesome feature of censored data is known as censored data analysis (CDA) methods. 

The first CDA method compared was casewise deletion, which simply deleted all nondetects from the analysis [39]. Casewise deletion remains widely used [39], even though exclusion of left-censored nondetects means elimination of the smallest values, which introduces upward bias into parameter estimates [39, 65, 66]. We also compared the effects of 3 CDA methods to replace nondetects with single numerical estimates. First were substitution methods, which replace all nondetects with an arbitrary constant (typically zero, some function of QL, or the mean) [62, 67]. Problems with substitution methods include the arbitrary choice of constant and underestimation of variability associated with the use of a constant rather than a randomly distributed variable [62]. Three substitution datasets were created by replacing each nondetect with a constant (zero, QL/2, and QL).

The second CDA method used maximum likelihood estimation (MLE) for single imputation. The mean (*x* = −0.485) and standard deviation (*s* = 0.910) of the lognormal distribution assumed to generate the censored hsCRP dataset were estimated from observed data [68, 69]. The assumption that the true underlying distribution of observed hsCRP values was lognormal [67, 68] was confirmed by the Anderson-Darling test and by coefficient-based tests [70]. Realistic values were simulated for all nondetects by random sampling from a lognormal distribution defined by the estimated parameters [69]. Simulated values below the QL were considered eligible as replacements and randomly substituted for all nondetects [11, 55, 71, 72]. 

The last CDA method used the NORM statistical software for multiple imputation (MI) [73]. MI datasets were formed by imputing probable values for all hsCRP nondetects, based on information derived from the variance/covariance matrix plus random error [74, 75]. Imputed values were only considered eligible substitutions for nondetects if they were less than the QL, in order to preserve the informative left-censoring mechanism, and were randomly substituted for nondetects. For data missing at random (non-informative censoring), as few as *m* = 5 imputed datasets may be needed [75, 76]. Due to the informative nature of left-censored data, we used more imputations (*m* = 13), as recommended [64, 77]. Regression coefficients and estimated group means were averaged across imputations, and within and between variance components estimated following Rubin's method [76, 78]. Results from the Kaplan-Meier (KM) non-parametric method [46] were treated as the standard of comparison for the other CDA methods. 

### 2.5. Reference Intervals

The KM approach to censored data does not substitute probable imputed values for nondetects but relies on analysis of the original censored dataset. Use of the casewise deletion dataset, in which all of the lowest values remain unobserved (nondetects), will yield biased reference intervals. Therefore, the substitution and imputation datasets were compared for their reliability in reference interval estimation. There are two common mistakes in estimating so-called “normal ranges” for clinical analytes. The first is to uncritically apply statistical methods based on the normal distribution to nonnormally distributed variables (biomarkers and other clinical analytes), rather than using appropriate methods from laboratory medicine [19–21, 31]. The second mistake is to assume that statistically significant covariates are automatically useful as clinical guidelines [19–21, 31]. Partitioning requires justification that the resulting subgroups have less reduced variability and narrower reference intervals relative to pooled data [31, 79, 80]. We used several guidelines to determine when to partition into subgroups. A *z* ^*^ statistic that exceeded the critical value of 5.0 was sufficient justification for partitioning. Alternately, a *z* ^*^ > 3.0 and either ≥10% reduction in subgroup standard deviations relative to pooled data, or a ratio of standard deviations ≥1.5, also justified partitioning [31, 79, 80]. Reference intervals were estimated using the non-parametric Harrell-Davis bootstrap for large (*n* ≥ 120) samples [81, 82] or the robust method for smaller samples [67, 83]. All calculations were performed on MedCalc version 12.2.1.0 [84].

## 3. Results

### 3.1. Nonrandom Missingness of hsCRP

We first tested for the presence of bias or nonrandom patterns of missing hsCRP data relative to the covariates (see Table 1). There was no association of hsCRP (observed, nondetects) with sex (*G* ^2^_1_ = 1.67, *P* = 0.197) or decade of life (*G* ^2^_1_ = 2.92, *P* = 0.232). hsCRP levels had a near-significant association with heart disease (*G* ^2^_1_ = 3.564, *P* = 0.059). There were significant associations between hsCRP and both HCV infection (*G* ^2^_1_ = 38.42, *P* < 0.000) and health status (*G* ^2^_1_ = 10.29, *P* = 0 = 0.013). These associations were quite strong. HCV-infected animals were 6.4 times more likely to have hsCRP nondetects, compared to uninfected animals (Little's MCAR *t* = 38.280, *P* < 0.000). Similarly, healthy animals were 2.9 times more likely to have hsCRP nondetects, compared to unhealthy animals (Little's MCAR *t* = 7.655, *P* = 0.022). These associations signaled a need to account for the effects of HCV infection and health status in subsequent analyses. 

### 3.2. Determinants of Serum hsCRP Level

We analyzed the different hsCRP datasets (casewise deletion, substitution, single MLE substitution, and multiple imputation) using the covariates age, sex, HCV infection, and cardiovascular disease. The casewise deletion dataset (*N* = 185) was smaller than all other CDA datasets (*N* = 257). Results of the Kaplan-Meier survival analysis were treated as the standard, against which the other strategies were compared. 

### 3.3. Kaplan-Meier

Neither sex (*X* ^2^_1_ = 1.56, *P* = 0.211) nor CVD status (*X* ^2^_1_ = 0.73, *P* = 0.393) had significant associations with hsCRP levels. As a quantitative covariate, age had a significant linear effect on hsCRP (*t* = 2.933, *P* = 0.003). Each year of life increased hsCRP by 0.027 mg/L. At that rate, a 10-year-old chimpanzee with 1.00 mg/L hsCRP would be expected to rise to 1.31 mg/L by the age of 20 yr. But when categorized by decade of life, the age trend failed to reach significance (*X* ^2^_2_ = 4.91, *P* = 0.086; 10–19 yr, 0.59 mg/L; 20–29 yr, 0.68 mg/L; 30+ yr, 0.76 mg/L; overall mean equaled 0.66 mg/L; see Table 2). Health had a modest effect (*X* ^2^_1_ = 5.051, *P* = 0.025), with healthy animals having lower median hsCRP (0.62 mg/L) than sick animals (0.79 mg/L; see Table 2). HCV infection strongly reduced hsCRP levels (*X* ^2^_1_ = 37.75, *P* < 0.000). Infected animals had an estimated median of 0.30 mg/L of hsCRP, compared to 0.79 mg/L for uninfected animals (Table 2). These KM results were taken as the standard for comparison of other CDA methods. 

### 3.4. Casewise Deletion

The casewise deletion dataset was analyzed next (*N* = 185). Neither sex nor health nor CVD status had any effect on hsCRP levels (Table 3). HCV status was highly significant (*F*_1,180_ = 11.074, *P* = 0.001; Table 3). Modeled as a quantitative covariate, age had a statistically significant effect of hsCRP (*F*_1,180_ = 6.624, *P* = 0.011) but its effect was relatively weak (*β*_Age_ = 1.02). A 10-year-old animal with 1.00 mg/L of hsCRP would be expected to rise to 1.22 mg/L by the age of 20 yr. When categorized by decade of life, there was an insignificant tendency for hsCRP to increase linearly across decades (*F*_1,183_ = 2.800, *P* = 0.096; see Table 3). Specifically, 10–19 yr olds had lower mean levels of hsCRP (0.91 mg/L) than 20–29 yr olds (1.02 mg/L), which were lower than the 30+ yr olds (1.14 mg/L). 

### 3.5. Substitution Methods

**Figure 2:**
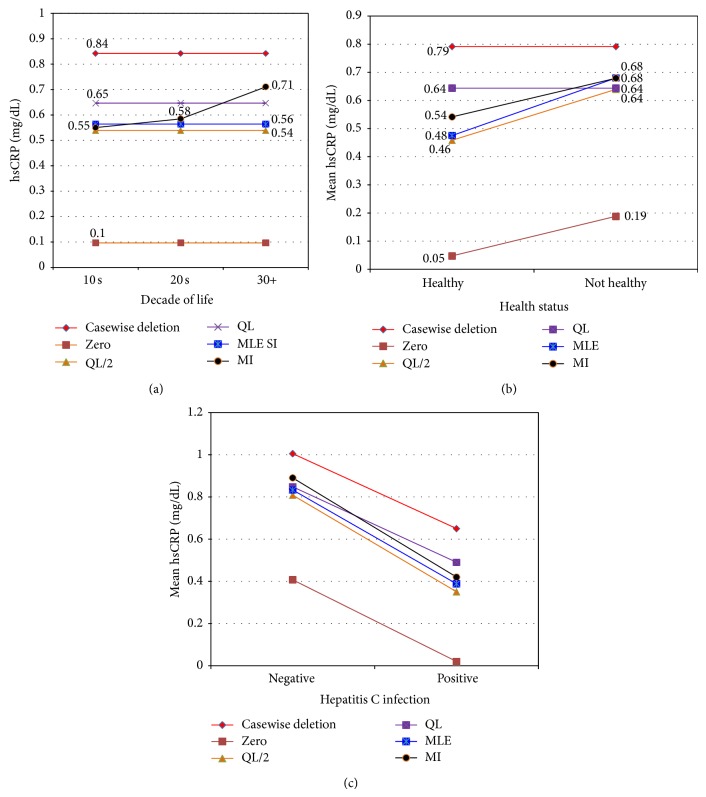
(a) Expected mean hsCRP levels by Decade of life, for all 6 datasets. (b) Expected mean hsCRP levels by Health Status, for all 6 datasets. (c) Expected mean hsCRP levels, by Hepatitis C infectious status, for all 6 datasets.

ANOVA analyses of the 3-substitution datasets (zero, QL/2, and QL) indicated agreement on lack of significance for sex and CVD and significant effects for HCV infection and age (Table 3). But results for health status varied by substitution model. Health was not significant for QL substitution, but it was significant for QL/2 and especially zero substitution (Table 3). The main difference between substitution models was that estimated effect sizes (group means) for age and HCV status reflected the size of the substitution constant (Figures 2(a) and 2(c); Tables 4 and 6). 

### 3.6. MLE Single Imputation

We then analyzed the MLE single imputation dataset. Results indicated that age, health, and HCV infection status all significantly influenced hsCRP levels (Table 3). But there was no effect of sex or heart disease (Table 3). 

### 3.7. Multiple Imputation

We then analyzed the MI datasets. Results indicated that neither sex nor CVD were associated with hsCRP levels (Table 3). However, hsCRP was significantly associated with HCV infection, health status, and decade of life (Table 3). 

### 3.8. Comparison of CDA Methods

Comparison of these analyses revealed several trends in the results of the different CDA methods. First, casewise deletion always yielded the largest estimated group means, while zero substitution always yielded the smallest (Figures 2(a), 2(b), and 2(c); Tables 4, 5, and 6). This was because exclusion of the smallest (left censored) values by casewise deletion inevitably introduced upward bias in estimated group means. Conversely, zero substitution introduced downward bias. The other CDA methods yielded group means intermediate to these extremes. Secondly, substitution methods introduced severe bias. Specifically, estimated group means from the substitution datasets were related to the magnitude of the substitution constant. For decade, health and HCV infection, estimated means were largest for QL, intermediate for QL/2, and lowest for zero substitution (Figures 2(a), 2(b), and 2(c); Tables 4, 5, and 6). Substitution methods also failed to distinguish different group means by decade of life, resulting instead in a constant mean (Table 4; Figure 2(a), flat horizontal lines). By contrast, MLE and MI detected more subtle differences among group means already identified by KM (Figures 2(a), 2(b), and 2(c), sloped lines across factor levels). And only MI detected the significant increase in hsCRP levels across decade of life (Figure 2(a); Table 4). Substitution methods performed erratically for health status. QL failed to detect significant group differences, but QL/2 and zero detected some differences (Figure 2(b); Table 5). Casewise deletion failed to detect a significant effect of health status, while MLE and MI detected large increases in hsCRP by health (Figure 2(b); Table 5). Finally, all CDA methods agreed in detection of lower levels of hsCRP in HCV+ animals (Figure 2(c); Table 6). This suggested that the effect of HCV infection on hsCRP was so strong that even biased methods could easily detect it. Note that the between-group difference was more or less constant, as indicated by parallel lines (Figure 2(c); Table 6), except MI, which detected a steeper drop in hsCRP for HCV+ animals. The real issue concerns the reliability of the inferred group means depending on the CDA method used, with substitution methods being particularly subject to bias. 

### 3.9. Association of hsCRP with Liver Enzymes

We then tested for an association of hsCRP with 2 standard enzymes used to examine hepatocyte damage, alanine aminotransferase (Alt), and alkaline phosphatase (Alp) [85]. hsCRP was trichotomized (nondetect, <1.0 mg/L, and ≥1.0 mg/L). Archival data on Alt and Alp levels in healthy adult chimpanzees were log_n_ transformed to induce normality, then trichotomized by tertiles (<89, 89–125.9, and ≥126 U/L). There was a strong inverse association between Alp and hsCRP levels (*G* ^2^_4_ = 11.02, *P* = 0.026). Specifically, a chimpanzee with sub-threshold levels of hsCRP was 1.8 times more likely to have high rather than low levels of Alp, compared to chimpanzees with high levels of hsCRP. Alt was likewise trichotomized (<34 U/L, 34–47.9, and ≥48 U/L) and similar results were obtained. There was a highly significant inverse association between Alt and hsCRP (*G* ^2^_4_ = 29.15, *P* < 0.000). Specifically, chimpanzees with sub-threshold levels of hsCRP were 11.8 times more likely to have high rather than low levels of Alt, compared to chimpanzees with high levels of hsCRP. 

The effects of HCV infection on the liver enzymes, Alp and Alt, were further analyzed with ANOVA. For Alp, neither sex nor age were significant as covariates (*F*_1,235_ = 1.284, *P* = 0.258; and *F*_1,235_ = 0.001, *P* = 0.970). But HCV infections was a powerful predictor of Alp levels (*F*_1,237_ = 13.884, *P* < 0.000). Chimpanzees with HCV infection averaged 128.8 U/mL of Alp, for an increase of 22% from average level for non-infected animals (105.6 U/mL). For Alt, age was not significant (*F*_1,235_ = 0.006, *P* = 0.938). There was a significant sex difference (*F*_1,236_ = 30.218, *P* < 0.000) with males having higher levels of Alt (55.0 U/mL) than females (43.9 U/mL). HCV infection was again a strong predictor of Alp levels (*F*_1,236_ = 156.182, *P* < 0.000). Chimpanzees with HCV infection averaged 66.3 U/mL of Alp, representing an increase of 82% from average levels for non-infected animals (36.5 U/mL). 

### 3.10. Reference Intervals

**Figure 3:**
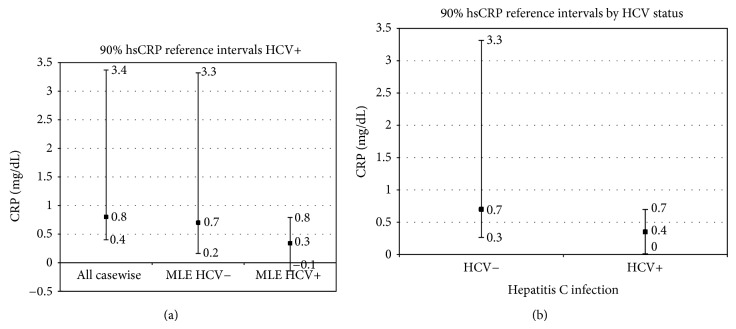
(a) 90% reference intervals, by HCV status (healthy adults, both sexes). (a) Casewise deletion and MLE reference intervals, by HCV status. (b) Multiple imputation reference intervals, by HCV status.

To determine justification for separate reference intervals, all statistically significant covariates in each CDA dataset (Table 3) were evaluated with partitioning tests (Table 7). For casewise deletion, the only significant covariate was HCV infection (Table 3). Results of the partitioning test did not suggest a need for separate reference intervals. Therefore, all observed data regardless of HCV infection status were used to construct a single 90% reference interval for all animals. This interval ranged from 0.40 mg/L to 3.37 mg/L, with a median of 0.80 mg/L (Figure 3(a)). 

For the substitution datasets, partitioning tests justified separate intervals by HCV status. For uninfected animals, the substitution datasets yielded medians (0.7 mg/L) and 95th percentiles (3.3 mg/L) identical to the casewise deletion and MLE intervals (Figure 3(a)). The only differences were in their lower (5%ile) boundaries, which varied according to the size of the substitution constant (0, 0.2, and 0.4). Substitution of a constant eliminated variation at the bottom of these distributions, so the robust method could not be used to estimate reference intervals for HCV-infected animals in the substitution datasets [80]. So reference intervals for the 3 substitution datasets were not presented. 

For the MLE dataset, HCV negative animals had an upper boundary (3.3 mg/L) and a median (0.7 mg/L) nearly identical to casewise deletion. But the bottom 5th percentile (0.2 mg/L) was considerably lower in MLE (0.2 mg/L) than casewise deletion (0.4 mg/L). For HCV-infected animals, use of the necessary robust procedure [80] with the small sample size resulted in a biologically impossible negative value (−0.1 mg/L) for the lower 5th percentile (Figure 3(a)). Both upper and lower MLE boundaries were more extreme than the MI intervals (Figure 3(a)). This was probably because random substitution used in MLE was unconstrained by the need to select statistically probable values based on the variance/covariance matrix, as implemented in the MI procedure. 

For the MI datasets, partitioning tests did not justify collapsing by health or decade of life, but HCV was highly significant (Table 7). The 90% reference interval for HCV uninfected chimpanzees was (0.3, 3.3) mg/L (Figure 3(b)). The 90% reference interval for HCV-infected chimpanzees was (0, 0.7) mg/L (Figure 3(b)). HCV-infected animals had median hsCRP levels (0.35 mg/L) half the size of uninfected animals (0.70 mg/L). The range of variation was 4 times greater among HCV non-infected than infected animals (Figure 3(b)). This may reflect the presence of other, as-yet unidentified conditions that influence hsCRP levels in uninfected animals, as in humans [55]. Median hsCRP level for HCV-negative animals (0.7 mg/L) was higher than the upper 90% boundary (0.3 mg/L) for HCV-positive animals, and equaled their 90th percentile. This small overlap indicated that hsCRP is considerably reduced in HCV-infected animals. In contrast to MLE, the MI dataset did not result in a negative lower boundary for HCV+ animals (Figures 3(a) and 3(b)). 

## 4. Discussion

hsCRP was of interest for its potential role as a biomarker of CVD and hepatic damage in chimpanzees. One problematic feature of these data was the presence of a moderate degree (28%) of censoring. The Kaplan-Meier nonparametric product-limit method for right censored survival data has well-understood properties but has seldom been applied to left-censored biomarker data [39, 47]. KM results showed that hsCRP levels were weakly associated with age, modestly associated with ill health, and strongly associated with hepatitis C infection. For age, there was a significant positive linear effect on hsCRP levels, and the trend was nearly significant when categorized by decade. For health status, sick animals had estimated median hsCRP (0.62 mg/L), 27% higher than healthy animals (0.79 mg/L). HCV-infected animals had estimated median hsCRP levels (0.3 mg/L) reduced by 62% relative to noninfected animals (0.79 mg/L). 

The KM results provided a standard for comparison of the performance of other CDA methods, all of which replaced nondetects with single specific values by different methods [76, 77]. Overall, the MI results most closely replicated the KM standard and also gave more precise results, such as group means, than KM. MLE single imputation gave reasonable results, even without utilizing information from the variance/covariance matrix, like MI. The casewise deletion dataset was highly upward biased, giving the largest estimated group means, while sometimes failing to detect significant effects. Inferences from the substitution datasets varied widely, making it difficult to identify any single “best” substitution constant. For example, only QL/2 identified the presence of a significant health effect known by KM results to exist (Tables 2 and 5). Furthermore, bias was evident in the group means estimated from all the substitution datasets, with the extent of bias determined by the size of the substitution constant. 

Regardless of CDA method, hsCRP was not associated with the presence of cardiovascular disease. This association has been observed repeatedly in prospective human studies [5, 7, 18, 53]. Its absence in chimpanzees was particularly surprising because chimpanzee CVD is characterized by myocardial fibrosis [22, 25] which is probably induced by hypertension [20, 21], and hsCRP is known to promote myocardial fibrosis [86]. The reason(s) for this lack of association in chimpanzees probably cannot be answered without detailed analysis of long-term (20+ years) followup data. Such prospective studies would also be needed to determine if hsCRP is also associated with non-vascular mortality, as observed in humans [53]. Also consistent with KM results, no other CDA method identified a sex difference. The lack of a sex difference in hsCRP levels has been repeatedly observed in human studies [55, 72, 87, 88]. 

The significant effect of health status on hsCRP in KM was identified in all but 2 CDA datasets (casewise deletion, QL). Health has also been shown to influence chimpanzee blood pressure [20, 21]. Interestingly, serum levels of hsCRP in healthy chimpanzees estimated by KM (0.62 mg/L) and MI (0.54 mg/L) were less than mean levels in healthy humans (0.8 mg/L; see also Table 5) [89]. 

For age, only MI identified the significant linear effect of age on hsCRP levels. For KM, this age trend declined to near-significance (*P* = 0.086) when categorized by decade, while for MI the decade effect remained significant (*P* = 0.045; Tables 2 and 3). The presence of a modest age effect has been reported in numerous human studies [35, 37, 72, 90–93]. This age trend was observed earlier in chimpanzees, although not reported as such. Reanalysis of published chimpanzee data showed near-significant effect of age on hsCRP levels (*G* ^2^_1_ = 3.47, *P* = 0.063), with subadults (<10 yo) 3.9 times more likely to have low (<1.0 mg/L) hsCRP levels than adults (≥10 yo;) [30]. Similarly, in the present study, 6 of 7 subadults excluded from analysis had sub-threshold levels of hsCRP. These observations suggest that elevated hsCRP may be a salient clinical characteristic of geriatric chimpanzees that deserves further study. 

All CDA methods confirmed the KM result that hsCRP levels declined with HCV infection. But effect sizes varied widely among methods. Estimated KM medians showed that HCV infection reduced serum hsCRP to less than half the level (0.30 mg/L) of uninfected animals (0.79 mg/L). These results were reasonably closely approximated by MLE (0.39 mg/L infected; 0.83 mg/L uninfected) and MI (0.42 mg/L infected, 0.89 mg/L uninfected). This difference, known in humans, has not previously been reported in chimpanzees [26–28, 89]. Contingency table analysis also indicated that HCV-infected animals were 6.4 times more likely to have sub-threshold hsCRP levels, relative to uninfected animals. These results indicated that reduced hsCRP levels (including nondetects) could be useful for monitoring and early detection of progressive liver dysfunction related to hepatitis C infection. Similar findings in humans led to a recommendation that separate reference intervals be estimated for HCV-infected individuals [28]. In humans, hsCRP levels initially rose in response to liver inflammation due to hepatitis infection, then declined with persistent infection [28, 89]. This pattern was thought to result from reduced CRP production in the liver secondary to progressive destruction of liver tissue [28, 89]. Analysis of 2 liver enzymes, Alt and Alp, demonstrated that liver dysfunction was associated with reduced serum hsCRP. High enzyme levels were associated with low hsCRP levels. These liver enzymes were also elevated by 22–82% in HCV-infected chimpanzees. The elevation of liver enzymes in HCV-infected animals, while hsCRP was reduced, suggested specific impairment of CRP synthesis, as in human studies [28, 89]. Thus, hsCRP appears to be an informative biomarker for long-term liver damage in HCV-infected chimpanzees. Confirmation of this etiology will require monitoring long-term (20+ yr) changes in hsCRP levels, across the course of hepatitis infection, plus postmortem evidence of hepatocellular damage. 

Overall, the 6 CDA methods for handling nondetects produced different outcomes, including statistical significance of covariates, estimated group means, and reference intervals. Compared to the KM standard, the best method was MI, which it most closely resembled. The worst method was casewise deletion, which gave highly biased results. Casewise deletion even failed to detect the powerful influence of HCV infection in reducing hsCRP levels, found in all other datasets. Consequently, the casewise deletion method should be avoided in biomarker analyses. Substitution methods performed poorly because they failed to identify significant covariates, while estimated group means depended strongly on the substitution constant used to replace nondetects. Such bias is a serious limitation, because estimation of effect sizes is the primary reason for use of statistical methods in the first place. Constant substitution methods should always be avoided in biomarker analysis. Finally, MLE performed better than casewise deletion and substitution methods but was less precise than MI for statistical inference. Although the values imputed by MLE derived probabilistically from an underlying lognormal distribution, they did not reflect probable values of hsCRP. That is because, unlike MI, MLE does not utilize information in the variance/covariance matrix. And MLE's reliance on a single imputed value fails to account for random variation in estimated nondetects [39]. Overall, MI gave results most similar to the KM standard. Although KM is a well-accepted method in survival analysis, it has not been used widely for biomarker analyses [39]. The primary limitations of KM are that estimated group means do not represent real effect sizes, because skew in the original censored data introduces bias, but medians may not be directly estimable depending on the pattern of censoring. Therefore, the best analytical method for handling left-censored biomarker data was MI. 

Regarding reference intervals, KM used the original left-censored data, equivalent to casewise deletion, which yielded upward biased reference intervals. Substitution methods biased the lower reference limit, with each lower limit equal to the substitution constant. Furthermore, substitution of a constant prevented use of the robust method for estimation of reference intervals for HCV-infected animals, which was another severe limitation of substitution methods. The MLE reference intervals were wider than the MI reference intervals, probably due to the inability of the MLE method to utilize information from the variance : covariance matrix. These limitations are further reasons to avoid casewise deletion, substitution, and MLE methods for biomarker analysis. For MI, the effect of decade of life was statistically significant but too small (0.08 mg/L/decade) to improve clinical precision and did not justify separate reference intervals. Separate reference intervals by HCV infection status were indicated in all datasets except casewise deletion. MI produced more reasonable intervals, with unbiased and positive lower boundaries (unlike casewise deletion, substitution, and MLE). Furthermore, the reduction in median hsCRP among infected (0.4 mg/L) versus uninfected chimpanzees (0.7 mg/L; Figure 3(b)) was comparable to the reduction observed among HCV-infected humans relative to healthy controls [28] that these differences were thought to result from cumulative liver damage from chronic HCV infection, [26–28, 50, 89] suggested the need to distinguish chimpanzee reference intervals based on HCV infection status. 

In summary, hsCRP data was moderately (27.9%) leftcensored. Missing data, although common, are not commonly reported. Standard reporting practices should include a description of the pattern and degree of missingness and the probable censoring mechanism, because different censoring mechanisms can bias results in different ways. Bias can also result from use of inappropriate analytical methods. Put another way, the presence of nondetects depends on the censoring mechanism, while the extent of bias depends on how missing data are handled in analysis. Casewise deletion ignores missing data and results in upward bias. Substitution methods introduce bias depending on the size of the substitution constant and should also be avoided. MLE-based single substitution was adequate for statistical inference but inadequate for reference intervals, because it gave biologically impossible negative values for lower boundaries. KM analysis using flipped censored data can be considered the analytical standard. But KM estimates of mean effects will be biased due to skew, while median effect sizes may not be estimable at all, depending on the pattern and extent of censoring. MI gave the best results for both statistical inference and reference intervals, although it was somewhat more complicated to implement. Overall, results demonstrated that hsCRP decreased significantly with HCV infection, increased moderately with health status, and increased modestly with age. We urge the use of KM or MI to handle left-censored biomarker data by other researchers, because other methods will yield biased and unreliable results. We recommend use of the hsCRP reference values estimated from the MI data (Figure 3(b)), as clinical guidelines for evaluating liver dysfunction in captive chimpanzees, in conjunction with liver enzymes, Alt and Alp. 

## Figures and Tables

**Table 1:** Classification of 258 hsCRP values on adult chimpanzees (≥10 yr old), by HCV status, health status, sex, and CRP missingness.

HCV status	Health status	Sex	hsCRP	
			Quantified	Nondetects
	Healthy	M	62	21
Uninfected	Healthy	F	64	13
	Unhealthy	M	19	0
	Unhealthy	F	16	2

	Healthy	M	9	21
Infected	Healthy	F	7	10
	Unhealthy	M	8	2
	Unhealthy	F	1	3

**Table 2:** Results of Kaplan-Meier analysis of flipped right censored data.

Factor	*X* ^2^	Estimated effect sizes		
		Factor level 1 (median)	Factor level 2 (median)	Factor level 3 (median)
HCV infection	37.746 (<0.000)∗∗	Infected 0.30	Not infected 0.79	—
Decade of life	4.910 (0.086)	10–19 yr 0.66	20–29 yr 0.66	30+ yr 0.66
Sex	1.561 (0.211)	Male 0.65	Female 0.65	—
Health status	5.051 (0.025)∗	Healthy 0.62	Unhealthy 0.79	—
CVD	0.730 (0.393)	No CVD 0.66	CVD 0.66	—

Tarone-Ware *X* ^2^ statistics (*P* value) for 5 covariates (HCV infection, decade of life, sex, health, and CVD). Data was the right censored “flipped” version of the original left-censored data. All *X* ^2^ tests were on 1 degree of freedom, except decade which used 2 degrees of freedom. Estimated medians were interpolated from the ranked KM results.

^*^ *P* < 0.05, ^**^ *P* < 0.01.

**Table 3:** ANOVA *F*-statistics (*P* values) for 5 covariates in 6 datasets.

Dataset	HCV infection	Decade of life^1^	Sex	Health status	CVD
Casewise deletion	11.074 (0.001)∗∗	2.800 (0.096)	0.013 (0.910)	0.217 (0.642)	1.385 (0.241)
QL	33.230 (<0.000)∗∗	2.211 (0.112)	0.078 (0.780)	2.601 (0.108)	0.881 (0.349)
QL/2	48.518 (<0.000)∗∗	1.733 (0.172)	0.279 (0.598)	6.630 (0.011)∗	0.327 (0.568)
Zero	50.860 (<0.000)∗∗	1.231 (0.294)	0.702 (0.403)	9.739 (0.002)∗∗	0.044 (0.834)
MLE	37.777 (<0.000)∗∗	1.333 (0.266)	0.062 (0.803)	7.284 (0.007)∗∗	0.739 (0.391)
MI	47.208 (<0.000)∗∗	3.738 (0.045)∗	0.068 (0.794)	4.083 (0.044)∗	0.513 (0.474)

*F*-statistics (*P* value) for 5 covariates (HCV infection, age by decade of life, sex, health, and cardiovascular disease), for each of 6 different datasets used to handle missing data. Datasets include casewise deletion (the original left-censored data with 72 sub-threshold nondetects), 3 substitution datasets [QL, QL/2 or Zero], MLE (a single imputation based on maximum likelihood estimation from a log-normal distribution), and MI (multiple imputation of estimates based on the variance-covariance matrix plus random error).

^*^ *P* < 0.05, ^**^ *P* < 0.01.

^1^A single degree of freedom linear contrast (−1 0 +1) was used to test the hypothesis that hsCRP increased linearly with decade of life (10–19 yr olds, 20–29 yr olds, and 30+ yr olds).

**Table 4:** Expected hsCRP level (mg/L) by decade of life, in 7 CDA datasets.

Dataset	10–19 yo	20–29 yo	30+ yo
K-M standard		0.66	
Casewise deletion		0.84	
Substitution: QL		0.65	
Substitution: QL/2		0.54	
Substitution: Zero		0.10	
MLE		0.56	
Multiple imputation	0.55	0.58	0.71

Expected level of hsCRP (mg/L) by decade of life. For datasets wherein decade of life was not statistically significant, group means were not relevant, cells were combined, and the single cell entry reflects the single overall mean. Number for the KM standard refers to estimated median level. All others refer to estimated means.

**Table 5:** Expected hsCRP level (mg/L), by health status, in 7 CDA datasets.

Dataset	Healthy	Not healthy
Kaplan-Meier standard	0.62	0.79
Casewise deletion	0.79	
Substitution: QL	0.64	
Substitution: QL/2	0.46	0.64
Substitution: Zero	0.05	0.19
MLE	0.48	0.68
Multiple imputation	0.54	0.68

Expected mean level of hsCRP (mg/L), by HCV infections status (infected or uninfected). Numbers for the KM standard refer to estimated median levels. All others refer to estimated means.

**Table 6:** Expected hsCRP level (mg/L), by HCV status, in 7 CDA datasets.

Dataset	HCV negative	HCV-infected
Kaplan-Meier standard	0.79	0.30
Casewise deletion	1.01	0.65
Substitution: QL	0.85	0.49
Substitution: QL/2	0.81	0.35
Substitution: Zero	0.41	0.02
MLE	0.83	0.39
Multiple imputation	0.89	0.42

Expected mean level of hsCRP (mg/L), by HCV infections status (not infected, infected). All datasets detected significant group differences, but resulted in different expected group means. Numbers for the KM standard refer to estimated median levels. All others refer to estimated means.

**Table:** (a) Health status

Dataset	*z* ^*^	*s*1/*s*2	*h*
Casewise deletion	2.05	1.07	0.8%
Zero	2.89	1.31	1.75
DL/2	2.08	1.05	0.9%
DL	n/a	n/a	n/a
MLE	2.39	1.15	1.2%
MI	n/a	n/a	n/a

**Table:** (b) Decade of life

Dataset	*z* ^*^	*s*1/*s*2	*h*
Casewise deletion	1.77	1.03	0.7%
Zero	n/a	n/a	n/a
DL/2	n/a	n/a	n/a
DL	n/a	n/a	n/a
MLE	n/a	n/a	n/a
MI	n/a	n/a	n/a

**Table:** (c) HCV infection status

Dataset	*z* ^*^	*s*1/*s*2	*h*
Casewise deletion	4.19	1.30	3.5%
Zero	6.31	1.17	7.4%
DL/2	7.57	1.31	10.2%
DL	7.29	1.74	9.5%
MLE	6.79	1.36	8.4%
MI	6.99	1.30	8.9%

Listed factors (health, decade, and HCV) were considered clinically significant and separate reference intervals constructed if *z* ^*^ > 5.0, *s*1/*s*2 > 1.50, or *h* > 10%. Only statistically significant covariates were tested (see Table 2). n/a means the factor was not significant and was not tested. See text for discussion.

## Abbreviations

ANOVA: Analysis of variance
CVD: Cardiovascular disease
CDA: Censored data analysis
hsCRP: High-sensitivity C-reactive protein
MLE: Maximum likelihood estimation
MI: Multiple imputation
Nt: Nucleotide position.
